# The mechanisms for pattern completion and pattern separation in the hippocampus

**DOI:** 10.3389/fnsys.2013.00074

**Published:** 2013-10-30

**Authors:** Edmund T. Rolls

**Affiliations:** ^1^Oxford Centre for Computational NeuroscienceOxford, UK; ^2^Department of Computer Science, University of WarwickCoventry, UK

**Keywords:** hippocampus, pattern separation, pattern completion, episodic memory, attractor network, pattern association network, competitive network, recall

## Abstract

The mechanisms for pattern completion and pattern separation are described in the context of a theory of hippocampal function in which the hippocampal CA3 system operates as a single attractor or autoassociation network to enable rapid, one-trial, associations between any spatial location (place in rodents, or spatial view in primates) and an object or reward, and to provide for completion of the whole memory during recall from any part. The factors important in the pattern completion in CA3 together with a large number of independent memories stored in CA3 include a sparse distributed representation which is enhanced by the graded firing rates of CA3 neurons, representations that are independent due to the randomizing effect of the mossy fibers, heterosynaptic long-term depression as well as long-term potentiation in the recurrent collateral synapses, and diluted connectivity to minimize the number of multiple synapses between any pair of CA3 neurons which otherwise distort the basins of attraction. Recall of information from CA3 is implemented by the entorhinal cortex perforant path synapses to CA3 cells, which in acting as a pattern associator allow some pattern generalization. Pattern separation is performed in the dentate granule cells using competitive learning to convert grid-like entorhinal cortex firing to place-like fields. Pattern separation in CA3, which is important for completion of any one of the stored patterns from a fragment, is provided for by the randomizing effect of the mossy fiber synapses to which neurogenesis may contribute, by the large number of dentate granule cells each with a sparse representation, and by the sparse independent representations in CA3. Recall to the neocortex is achieved by a reverse hierarchical series of pattern association networks implemented by the hippocampo-cortical backprojections, each one of which performs some pattern generalization, to retrieve a complete pattern of cortical firing in higher-order cortical areas.

## Introduction

There is great interest in how pattern separation and pattern completion in the hippocampus contribute to its functions in memory and spatial function (Rolls and Treves, 1998; Nakazawa et al., 2002, 2003; Wills et al., 2005; Rolls and Kesner, 2006; Kesner, 2007, 2013; Leutgeb et al., 2007; McHugh et al., 2007; Hunsaker and Kesner, 2008, 2013; Giocomo et al., 2011; Jezek et al., 2011; Kesner et al., 2012; Nakashiba et al., 2012).

The aim of this paper is to describe some of the different types of pattern separation and pattern completion in the hippocampal system, and the mechanisms that implement them. It is important to appreciate that there are different mechanisms each of which contributes to pattern separation or pattern completion in the hippocampal system, for this helps not only in the understanding of how the hippocampal system operates, but also helps in the evaluation of the effects of changes that influence each of these mechanisms. These different mechanisms are separated into different subsections of this paper, so that the operation and contributions of each mechanism can be clarified and evaluated. The different mechanisms for pattern separation and pattern completion are considered in the context of a theory of hippocampal function (Rolls, 2008, 2010b). More comprehensive descriptions of this theory of hippocampal function, and of differences between the primate and rodent hippocampal neuronal representations and the implications for understanding human memory, are provided elsewhere (Rolls and Kesner, 2006; Rolls and Xiang, 2006; Rolls, 2008, 2010b, 2013). The theory has been developed through many stages (Rolls, 1987, 1989a,b,c, 1990a,b, 1991, 1995, 1996b, 2008, 2010b; Treves and Rolls, 1991, 1992, 1994; Rolls and Treves, 1998; Rolls and Kesner, 2006; Rolls and Deco, 2010), has as a predecessor developments made by David Marr (1971) (though he never identified the CA3 system as an autoassociation network), and has benefitted greatly from collaborations with many whose names appear below in the citations, including Alessandro Treves and Simon Stringer. The operation of pattern association networks, autoassociation networks, and competitive networks has been described elsewhere (Hertz et al., 1991; Rolls and Treves, 1998; Rolls, 2008).

## Background to the approach to hippocampal function

### Event and episodic memory

The focus is on a fundamental property of episodic memory, the ability to store and retrieve the memory of a particular single event involving an association between items such as the place and the object or reward seen at that place. Episodic memory in the sense of a series of linked events requires this type of event memory, and could be implemented by linking together a series of events.

An event consists of a set of items that occur together, such as seeing a particular object or person's face in a particular place. An everyday example might be remembering where one was for dinner, who was present, what was eaten, what was discussed, and the time at which it occurred. The spatial context is almost always an important part of an episodic memory (Dere et al., 2008), and it may be partly for this reason that episodic memory is linked to the functions of the hippocampal system, which is involved in spatial processing and memory. The ability to recall a whole memory from a partial cue is an important property of episodic memory, and is referred to as completion.

### Systems-level functions and connections of the primate hippocampus

Any theory of the hippocampus must state at the systems level what is computed by the hippocampus. Some of the relevant evidence about the functions of the hippocampus in memory comes from the effects of damage to the hippocampus, the responses of neurons in the hippocampus during behavior, and the systems-level connections of the hippocampus, described in more detail elsewhere (Rolls and Kesner, 2006; Rolls and Xiang, 2006; Rolls, 2008, 2010b). Many of the memory functions are important in event or episodic memory, in which the ability to remember what happened where on typically a single occasion (or trial in a learning experiment) is important. It is suggested that an autoassociation memory implemented by the CA3 neurons enables event or episodic memories to be formed by enabling associations to be formed between spatial and other including object or reward representations, and for completion to then occur in recall. An important property of this autoassociation and completion is that completion of a whole memory can occur from any part. This is different from pattern association memory, in which a visual stimulus might become associated with a taste by associative synaptic modification. Later presentation of the visual stimulus would retrieve the taste representation. However, presentation of the taste would not retrieve the visual representation, and this is an important and fundamental difference between autoassociation and pattern association, as described in detail elsewhere (Rolls and Treves, 1998; Rolls, 2008, 2014a).

**Figure 1:**
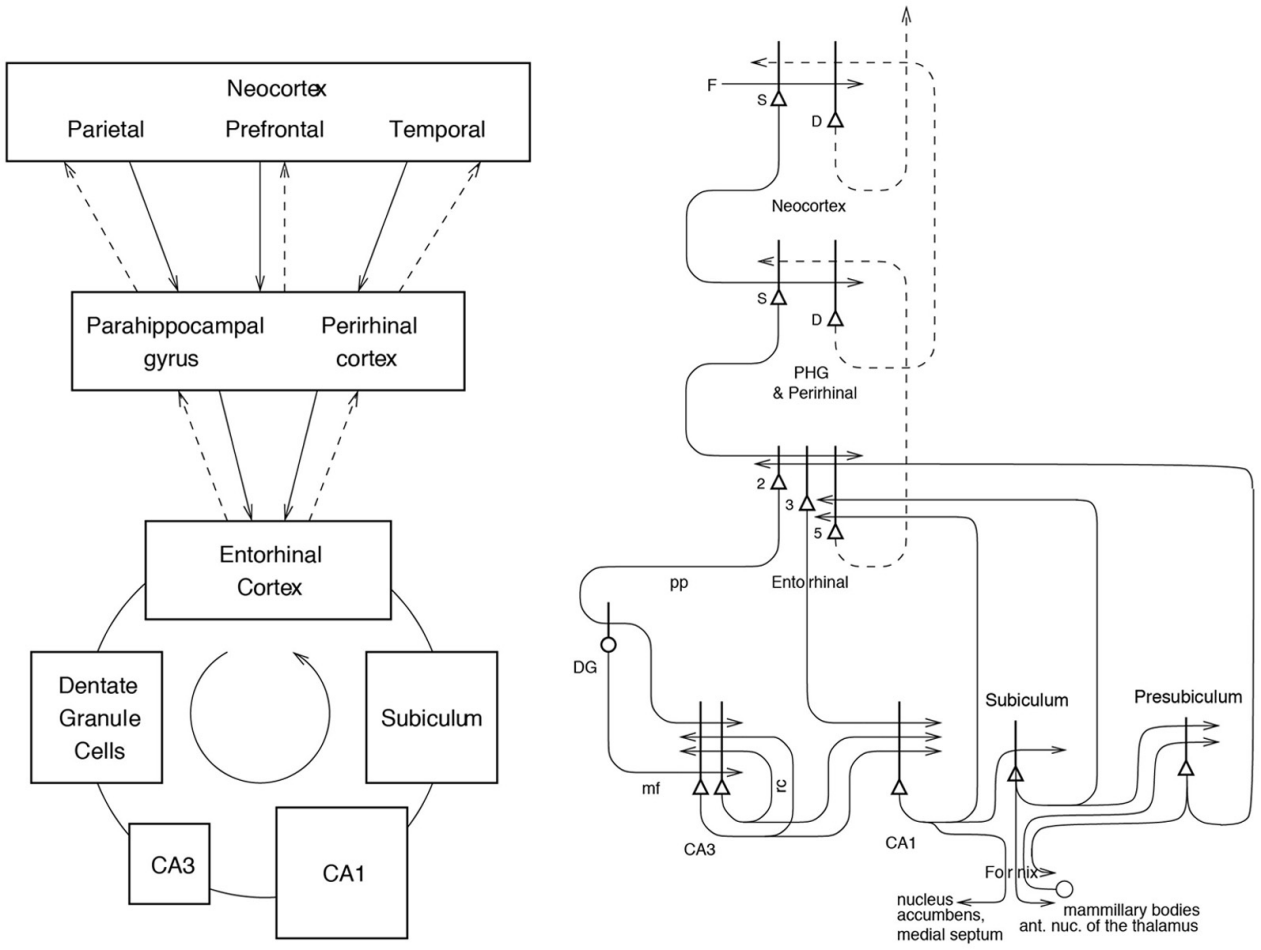
**Forward connections (solid lines) from areas of cerebral association neocortex via the parahippocampal gyrus and perirhinal cortex, and entorhinal cortex, to the hippocampus; and backprojections (dashed lines) via the hippocampal CA1 pyramidal cells, subiculum, and parahippocampal gyrus to the neocortex**. There is great convergence in the forward connections down to the single network implemented in the CA3 pyramidal cells; and great divergence again in the backprojections. **Left**: block diagram. **Right**: more detailed representation of some of the principal excitatory neurons in the pathways. Abbreviations: D, deep pyramidal cells; DG, dentate granule cells; F, forward inputs to areas of the association cortex from preceding cortical areas in the hierarchy; mf, mossy fibres; PHG, parahippocampal gyrus and perirhinal cortex; pp. perforant path; rc, recurrent collateral of the CA3 hippocampal pyramidal cells; S, superficial pyramidal cells; 2, pyramidal cells in layer 2 of the entorhinal cortex; 3, pyramidal cells in layer 3 of the entorhinal cortex. The thick lines above the cell bodies represent the dendrites.

Information stored in the hippocampus will need to be retrieved and affect other parts of the brain in order to be used. The information about episodic events recalled from the hippocampus could be used to help form semantic memories (Rolls, 1989b,c, 1990a; Treves and Rolls, 1994). For example, remembering many particular journeys could help to build a geographic cognitive map in the neocortex. The hippocampus and neocortex would thus be complementary memory systems, with the hippocampus being used for rapid, “on the fly,” unstructured storage of information involving activity potentially arriving from many areas of the neocortex; while the neocortex would gradually build and adjust on the basis of much accumulating information, often recalled from the hippocampal unstructured store, the semantic representation (Rolls, 1989b; Treves and Rolls, 1994; McClelland et al., 1995; Moscovitch et al., 2005). The theory shows how information could be retrieved within the hippocampus, and how this retrieved information could enable the activity in neocortical areas that was present during the original storage of the episodic event to be reinstated, thus implementing recall, by using hippocampo-neocortical backprojections as described elsewhere (Treves and Rolls, 1994; Rolls, 1995, 1996b, 2008, 2010b)(see Figure 1).

To understand the functions of the primate hippocampus in event or episodic memory, it is necessary to understand which other parts of the brain it receives information from. Does it for example receive object as well as spatial information as indicated by its anatomical connectivity? The primate hippocampus receives inputs via the entorhinal cortex (area 28) and the highly developed parahippocampal gyrus (areas TF and TH) as well as the perirhinal cortex from the ends of many processing streams of the cerebral association cortex, including the visual and auditory temporal lobe association cortical areas, the prefrontal cortex, and the parietal cortex (Van Hoesen, 1982; Amaral, 1987; Amaral et al., 1992; Suzuki and Amaral, 1994b; Witter et al., 2000b; Lavenex et al., 2004; Rolls and Kesner, 2006; Rolls, 2008) (see Figure 1). The hippocampus is thus by its connections potentially able to associate together object and spatial representations. In addition, the entorhinal cortex receives inputs from the amygdala, and the orbitofrontal cortex, which could provide reward-related information to the hippocampus (Suzuki and Amaral, 1994a; Carmichael and Price, 1995; Stefanacci et al., 1996; Pitkanen et al., 2002).

The primary output from the hippocampus to neocortex originates in CA1 and projects to subiculum, entorhinal cortex, and parahippocampal structures (areas TF-TH) as well as prefrontal including orbitofrontal cortex (Van Hoesen, 1982; Witter, 1993; Delatour and Witter, 2002; Van Haeften et al., 2003) (see Figure 1), though there are other outputs (Rolls and Kesner, 2006; Rolls, 2014b). These are the pathways that are likely to be involved in the retrieval of information from the hippocampus back to the neocortex.

The theory is a quantitative theory, and the numbers of synapses on the different types of neuron is an important feature of the circuitry emphasized next.

### Hippocampal circuitry

Hippocampal circuitry (Amaral and Witter, 1989; Storm-Mathiesen et al., 1990; Amaral, 1993; Witter et al., 2000b; Naber et al., 2001; Lavenex et al., 2004; Andersen et al., 2007; Witter, 2007; Kondo et al., 2009) is illustrated in Figure 1.

**Figure 2:**
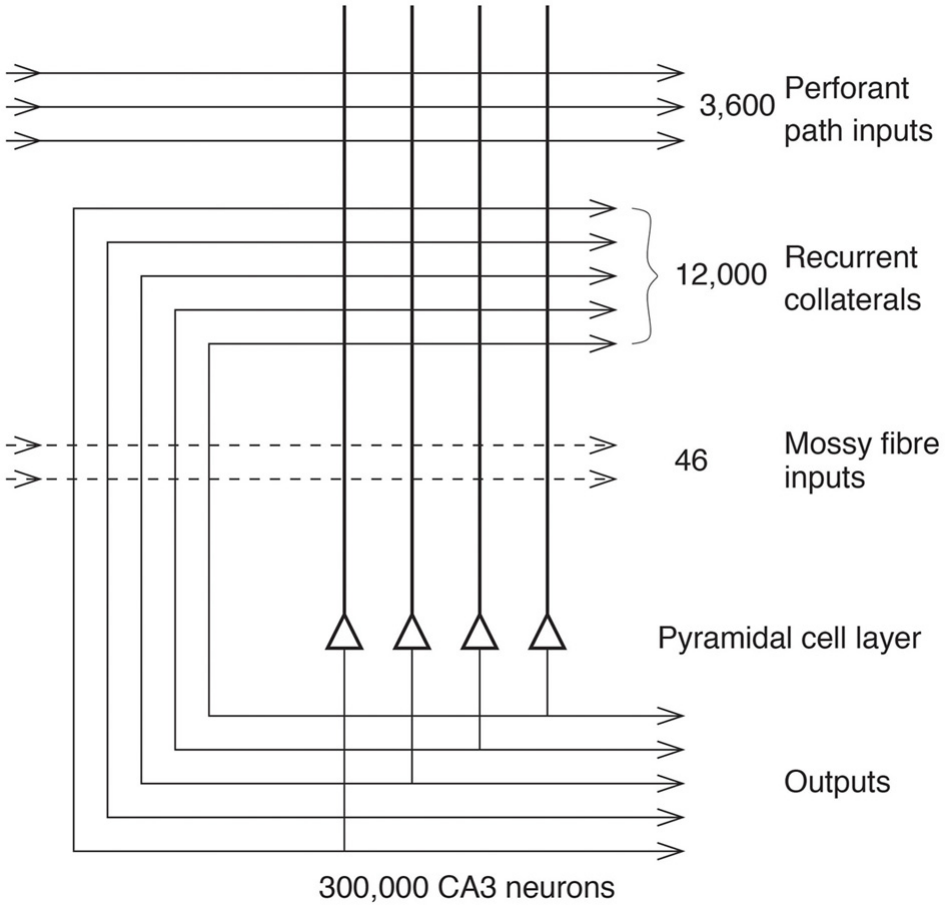
**The numbers of connections from three different sources onto each CA3 cell from three different sources in the rat**. (After Treves and Rolls, 1992; Rolls and Treves, 1998).

Projections from the entorhinal cortex layer 2 reach the granule cells (of which there are 10^6^ in the rat) in the dentate gyrus (DG), via the perforant path (pp) (Witter, 1993). The granule cells project to CA3 cells via the mossy fibres (mf), which provide a *sparse* but powerful connection to the 3 × 10^5^ CA3 pyramidal cells in the rat. Each CA3 cell receives approximately 46 mossy fiber inputs, so that the sparseness (or dilution) of this connectivity is thus 0.000046. By contrast, there are many more—possibly weaker—direct perforant path inputs also from layer 2 of the entorhinal cortex onto each CA3 cell, in the rat of the order of 3600. The largest number of synapses (about 1.2 × 10^4^ in the rat) on the dendrites of CA3 pyramidal cells is, however, provided by the (recurrent) axon collaterals of CA3 cells themselves (rc) (see Figure 2). It is remarkable that the recurrent collaterals (RCs) are distributed to other CA3 cells largely throughout the hippocampus (Amaral and Witter, 1989, 1995; Amaral et al., 1990; Ishizuka et al., 1990; Witter, 2007), so that effectively the CA3 system provides a single network, with a connectivity of approximately 2% between the different CA3 neurons given that the connections are bilateral. The CA3-CA3 RC system is even more extensive in macaques than in rats (Kondo et al., 2009). The neurons that comprise CA3, in turn, project to CA1 neurons via the Schaffer collaterals. In addition, projections that terminate in the CA1 region originate in layer 3 of the entorhinal cortex (see Figure 1).

## CA3 as an autoassociation or attractor memory: pattern completion

### Arbitrary associations, and pattern completion in recall

Many of the synapses in the hippocampus show associative modification as shown by long-term potentiation, and this synaptic modification appears to be involved in learning (see Morris, 1989, 2003; Morris et al., 2003; Nakazawa et al., 2003, 2004; Lynch, 2004; Andersen et al., 2007; Wang and Morris, 2010; Jackson, 2013). On the basis of the evidence summarized above, Rolls (1987, 1989a,b,c, 1990a,b, 1991) and others (McNaughton and Morris, 1987; Levy, 1989; McNaughton, 1991) have suggested that the CA3 stage acts as an autoassociation memory which enables episodic memories to be formed and stored in the CA3 network, and that subsequently the extensive RC connectivity allows for the retrieval of a whole representation to be initiated by the activation of some small part of the same representation (the recall cue). The crucial synaptic modification for this is in the RC synapses. [A description of the operation of autoassociative networks is provided in detail elsewhere (Amit, 1989; Hertz et al., 1991; Rolls and Treves, 1998; Rolls and Deco, 2002, 2010; Rolls, 2010a) including *Memory, Attention, and Decision-Making* (Rolls, 2008)].

The architecture of an autoassociation network is effectively that of the RC synapses shown in Figure 2, and the learning rule for the change in the synaptic weights is as shown in Equation (1) (Rolls and Treves, 1998; Rolls, 2008).

(1) $$\delta w_{ij}=k\;.\;r_{i}\;.\;{r^{\prime }}_{j}$$

where *k* is a constant, *r*_*i*_ is the activation of the dendrite (the postsynaptic term), *r*′_*j*_ is the presynaptic firing rate, and δ*w*_*ij*_ is the change in the synaptic weight *w*_*ij*_. (*w*_*ij*_ refers to the *j*'th synapse onto the *i*'th neuron. An introduction to autoassociation, competitive, and pattern association networks is provided in the Appendices of *Memory, Attention and Decision-Making: A Unifying Computational Neuroscience Approach* Rolls, 2008.)

The hypothesis is that because the CA3 operates effectively as a single network, it can allow arbitrary associations between inputs originating from very different parts of the cerebral cortex to be formed. These might involve associations between information originating in the temporal visual cortex about the presence of an object, and information originating in the parietal cortex about where it is. I note that although there is some spatial gradient in the CA3 recurrent connections, so that the connectivity is not fully uniform (Ishizuka et al., 1990; Witter, 2007), nevertheless the network will still have the properties of a single interconnected autoassociation network allowing associations between arbitrary neurons to be formed, given the presence of many long-range connections which overlap from different CA3 cells, and the ability of attractor networks to operate with diluted connectivity shown in our computational studies prompted by this issue (Treves, 1990; Treves and Rolls, 1991; Rolls, 1995, 2012a; Rolls and Webb, 2012). It is very interesting indeed that in primates (macaques), the associational projections from CA3 to CA3 travel extensively along the longitudinal axis, and overall the radial, transverse, and longitudinal gradients of CA3 fiber distribution, clear in the rat, are much more subtle in the non-human primate brain (Kondo et al., 2009). The implication is that in primates, the CA3 network operates even more as a single network than in rodents.

Crucial issues include how many memories could be stored in this system (to determine whether the autoassociation hypothesis leads to a realistic estimate of the number of memories that the hippocampus could store); whether the whole of a memory could be completed from any part; whether the autoassociation memory can act as a short term memory, for which the architecture is inherently suited; and whether the system could operate with spatial representations, which are essentially continuous because of the continuous nature of space. These and related issues are considered in the remainder of section Storage Capacity and in more detail elsewhere (Rolls and Kesner, 2006; Rolls, 2008).

### Storage capacity

We have performed quantitative analyses of the storage and retrieval processes in the CA3 network (Treves and Rolls, 1991, 1992; Webb et al., 2011; Rolls, 2012a; Rolls and Webb, 2012). We have extended previous formal models of autoassociative memory (see Amit, 1989) by analyzing a network with graded response units, so as to represent more realistically the continuously variable rates at which neurons fire, and with incomplete connectivity (Treves, 1990; Rolls, 1991; Treves and Rolls, 1991; Rolls et al., 1997b; Webb et al., 2011; Rolls and Webb, 2012). We have found that in general the maximum number *p*_max_ of firing patterns that can be (individually) retrieved is proportional to the number *C*^RC^ of (associatively) modifiable RC synapses on to each neuron, by a factor that increases roughly with the inverse of the sparseness *a* of the neuronal representation. [Each memory is precisely defined in the theory: it is a set of firing rates of the population of neurons (which represent a memory) that can be stored and later retrieved, with retrieval being possible from a fraction of the originally stored set of neuronal firing rates.] The neuronal population sparseness *a* of the representation can be measured by extending the binary notion of the proportion of neurons that are firing to any one stimulus or event as

(2) $$a={\left(\sum_{i\;=\;1,n}r_{i}/N\right)}^{2}/\sum_{i\;=\;1,n}\left(r_{i}^{2}/N\right)$$

where *r*_*i*_ is the firing rate of the *i*'th neuron in the set of *N* neurons. The sparseness ranges from 1/*N*, when only one of the neurons responds to a particular stimulus (a local or grandmother cell representation), to a value of 1.0, attained when all the neurons are responding to a given stimulus. Approximately,

(3) $$p_{\text{max}}≅\frac{C^{\text{RC}}}{\left[a\ln(1/a)\right]}k$$

where *k* is a factor that depends weakly on the detailed structure of the rate distribution, on the connectivity pattern, etc., but is roughly in the order of 0.2-0.3 (Treves and Rolls, 1991). For example, for *C*^RC^ = 12,000 and *a* = 0.02, *p*_max_ is calculated to be approximately 36,000. This analysis emphasizes the utility of having a sparse representation in the hippocampus, for this enables many different memories to be stored. [The sparseness *a* in this equation is strictly the population sparseness (Treves and Rolls, 1991; Franco et al., 2007). The population sparseness *a*^p^ would be measured by measuring the distribution of firing rates of all neurons to a single stimulus at a single time. The single neuron sparseness or selectivity *a*^s^ would be measured by the distribution of firing rates to a set of stimuli, which would take a long time. The selectivity or sparseness *a*^s^ of a single neuron measured across a set of stimuli often takes a similar value to the population sparseness *a*^p^ in the brain, and does so if the tuning profiles of the neurons to the set of stimuli are uncorrelated (Franco et al., 2007). These concepts are elucidated by Franco et al. (2007).] (I note that the sparseness estimates obtained by measuring early gene changes, which are effectively population sparsenesses, would be expected to depend greatly on the range of environments or stimuli in which these were measured. If the environment was restricted to one stimulus, this would reflect the population sparseness. If the environment was changing, the measure from early gene changes would be rather undefined, as all the populations of neurons activated in an undefined number of testing situations would be likely to be activated.)

In order for most associative networks to store information efficiently, heterosynaptic Long Term Depression (as well as LTP) is required (Rolls and Treves, 1990, 1998; Treves and Rolls, 1991; Fazeli and Collingridge, 1996; Rolls and Deco, 2002; Rolls, 2008). Simulations that are fully consistent with the analytic theory are provided by Rolls (1995, 2012a); Simmen et al. (1996) and Rolls et al. (1997b).

A number of points that arise, including measurement of the total amount of information (in bits per synapse) that can be retrieved from the network, the computational definition of a memory, the computational sense in which CA3 is an attractor network, and the possible computational utility of memory reconsolidation, are treated elsewhere (Rolls and Kesner, 2006; Rolls, 2008). Here I note that given that the memory capacity of the hippocampal CA3 system is limited, it is necessary to have some form of forgetting in this store, or other mechanism to ensure that its capacity is not exceeded. (Exceeding the capacity can lead to a loss of much of the information retrievable from the network.) Heterosynaptic LTD could help this *forgetting*, by enabling new memories to overwrite old memories (Rolls, 1996a, 2008). The limited capacity of the CA3 system does also provide one of the arguments that some transfer of information from the hippocampus to neocortical memory stores may be useful (see Treves and Rolls, 1994). Given its limited capacity, the hippocampus might be a useful store for only a limited period, which might be in the order of days, weeks, or months. This period may well depend on the acquisition rate of new episodic memories. If the animal were in a constant and limited environment, then as new information is not being added to the hippocampus, the representations in the hippocampus would remain stable and persistent. These hypotheses have clear experimental implications, both for recordings from single neurons and for the gradient of retrograde amnesia, both of which might be expected to depend on whether the environment is stable or frequently changing. They show that the conditions under which a gradient of retrograde amnesia might be demonstrable would be when large numbers of new memories are being acquired, not when only a few memories (few in the case of the hippocampus being less than a few hundred) are being learned.

### Recall and completion

A fundamental property of the autoassociation model of the CA3 RC network is that the recall can be symmetric, that is, the whole of the memory can be retrieved and completed from any part (Rolls and Treves, 1998; Rolls and Kesner, 2006; Rolls, 2008). For example, in an object-place autoassociation memory, an object could be recalled from a place retrieval cue, and vice versa. In a test of this, Day, Langston and Morris (2003) trained rats in a study phase to learn in one trial an association between two flavors of food and two spatial locations. During a recall test phase they were presented with a flavor which served as a cue for the selection of the correct location. They found that injections of an NMDA receptor blocker (AP5) or AMPA/kainate receptor blocker (CNQX) to the dorsal hippocampus prior to the study phase impaired encoding, but injections of AP5 prior to the test phase did not impair the place recall, whereas injections of CNQX did impair the place recall. The interpretation is that somewhere in the hippocampus NMDA receptors are necessary for learning one-trial odor-place associations, and that recall can be performed without further involvement of NMDA receptors.

Evidence that the CA3 system is not necessarily required during recall in a reference memory spatial task, such as the water maze spatial navigation for a single spatial location task, is that CA3 lesioned rats are not impaired during recall of a previously learned water maze task (Brun et al., 2002; Florian and Roullet, 2004). However, if completion from an incomplete cue is needed, then CA3 NMDA receptors are necessary (presumably to ensure satisfactory CA3-CA3 learning) even in a reference memory task (Nakazawa et al., 2002; Gold and Kesner, 2005). Thus, the CA3 system appears to be especially needed in rapid, one-trial object-place recall, and when completion from an incomplete cue is required (see further section Pattern Separation Performed by Dentate Granule Cells).

Especially important though in assessing the implications of all such tests is that the theory sets out how the system operates when large numbers of memories, in the order of thousands, are to be stored and retrieved, and this is difficult to test adequately in behavioral experiments. Effects found when the storage and retrieval of just a few memories are tested may not reflect well the operation of the system when it is heavily loaded, as it is expected to be when operating in the natural environment.

Evidence for pattern completion has been observed using imaging with voltage-sensitive dye in the CA3 region of a rat hippocampal slice. Following the induction of long-term potentiation from two stimulation sites activated simultaneously, stimulation at either of the two sites produced the whole pattern of activation that could be produced from both stimulation sites before LTP, thus demonstrating pattern completion in CA3 (Jackson, 2013).

### Continuous, spatial, patterns and CA3 representations

The fact that spatial patterns, which imply continuous representations of space, are represented in the hippocampus has led to the application of continuous attractor models to help understand hippocampal function. This has been necessary, because space is inherently continuous, because the firing of place and spatial view cells is approximately Gaussian as a function of the distance away from the preferred spatial location, because these cells have spatially overlapping fields, and because the theory is that these cells in CA3 are connected by Hebb-modifiable synapses. This specification would inherently lead the system to operate as a continuous attractor network. Continuous attractor network models have been studied by Amari (1977); Zhang (1996); Samsonovich and McNaughton (1997); Battaglia and Treves (1998); Taylor (1999); Stringer and Rolls (2002); Stringer et al. (2002a,b, 2004) and Rolls and Stringer (2005) [see Rolls (2008)], and are described briefly next.

A “Continuous Attractor” neural network (CANN) can maintain the firing of its neurons to represent any location along a continuous physical dimension such as spatial view, spatial position, head direction, etc. It uses excitatory RC connections between the neurons (as are present in CA3) to reflect the distance between the neurons in the state space of the animal (e.g., place or head direction). These networks can maintain the bubble or packet of neural activity constant for long periods wherever it is started to represent the current state (head direction, position, etc.) of the animal, and are likely to be involved in many aspects of spatial processing and memory, including spatial vision. Global inhibition is used to keep the number of neurons in a bubble or packet of actively firing neurons relatively constant, and to help to ensure that there is only one activity packet.

**Figure 3:**
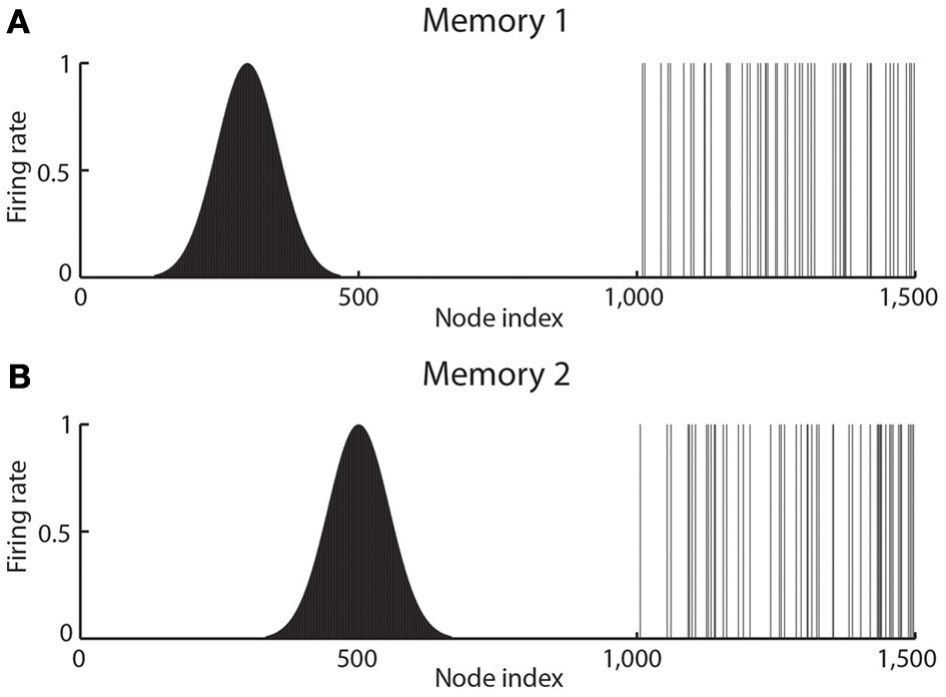
**The types of firing patterns stored in continuous attractor networks are illustrated for the patterns present on neurons 1–1000 for Memory 1 (when the firing is that produced when the spatial state represented is that for location 300), and for Memory 2 (when the firing is that produced when the spatial state represented is that for location 500)**. The continuous nature of the spatial representation results from the fact that each neuron has a Gaussian firing rate that peaks at its optimal location. This particular mixed network also contains discrete representations that consist of discrete subsets of active binary firing rate neurons in the range 1001–1500. The firing of these latter neurons can be thought of as representing the discrete events that occur at the location. Continuous attractor networks by definition contain only continuous representations, but this particular network can store mixed continuous and discrete representations, and is illustrated to show the difference of the firing patterns normally stored in separate continuous attractor and discrete attractor networks. For this particular mixed network, during learning, Memory 1 is stored in the synaptic weights **(A)**, then Memory 2 **(B)**, etc., and each memory contains part that is continuously distributed to represent physical space, and part that represents a discrete event or object.

Continuous attractor networks can be thought of as very similar to autoassociation or discrete attractor networks (Rolls, 2008), and have the same architecture. The main difference is that the patterns stored in a CANN are continuous patterns, with each neuron having broadly tuned firing which decreases with for example a Gaussian function as the distance from the optimal firing location of the cell is varied, and with different neurons having tuning that overlaps throughout the space. Such tuning is illustrated in Figure 3. For comparison, autoassociation networks normally have discrete (separate) patterns (each pattern implemented by the firing of a particular subset of the neurons), with no continuous distribution of the patterns throughout the space (see Figure 3). A consequent difference is that the CANN can maintain its firing at any location in the trained continuous space, whereas a discrete attractor or autoassociation network moves its population of active neurons toward one of the previously learned attractor states, and thus implements the recall of a particular previously learned pattern from an incomplete or noisy (distorted) version of one of the previously learned patterns.

Space is continuous, and object representations are discrete. If these representations are to be combined in for example an object-place memory, then we need to understand the operation of networks that combine these representations. Rolls et al. (2002) have shown that attractor networks can store both continuous patterns and discrete patterns (as illustrated in Figure 3), and can thus be used to store for example the location in (continuous, physical) space (e.g., the place “out there” in a room represented by spatial view cells) where an object (a discrete item) is present. We showed this by storing associated continuous and discrete representations in the same single attractor network, and then showing that the representation in the continuous space could be retrieved by the discrete object that was associated with that spatial position; and that the representation of the discrete object could be retrieved by providing the position in the continuous representation of space.

If spatial representations are stored in the hippocampus, the important issue arises in terms of understanding memories that include a spatial component or context of how many such spatial representations could be stored in a continuous attractor network. The very interesting result is that because there are in general low correlations between the representations of places in different maps or charts (where each map or chart might be of one room or locale), very many different maps or charts can be simultaneously stored in a continuous attractor network (Battaglia and Treves, 1998).

We have considered how spatial representations could be stored in continuous attractor networks, and how the activity can be maintained at any location in the state space in a form of short-term memory when the external (e.g., visual) input is removed. However, a property of some spatial representations is that they can be updated by self-motion, idiothetic, input, and mechanisms have been proposed for how this could be achieved (Samsonovich and McNaughton, 1997; Stringer et al., 2002a,b, 2005; Rolls and Stringer, 2005; Stringer and Rolls, 2006; Walters et al., 2013), including in the entorhinal cortex grid cell system (Kropff and Treves, 2008; Giocomo et al., 2011; Zilli, 2012). The ways in which path integration could be implemented in recurrent networks such as the CA3 system in the hippocampus or in related systems are described elsewhere (Samsonovich and McNaughton, 1997; Stringer et al., 2002a,b; McNaughton et al., 2006), and have been applied to primate spatial view cells by Rolls and colleagues (Stringer et al., 2004, 2005; Rolls and Stringer, 2005). Cognitive maps (O'Keefe and Nadel, 1978) can be understood by the operations of these attractor networks, and how they are updated by learning and by self-motion (Rolls, 2008). It has been argued that the bumpiness of the CA3 representation of space is more consistent with episodic memory storage, as argued in this paper, than with spatial path integration using the CA3 system as a continuous attractor network implementing path integration (Cerasti and Treves, 2013; Stella et al., 2013).

### Perforant path inputs to CA3 cells initiate recall in CA3 and contribute to generalization

By calculating the amount of information that would end up being carried by a CA3 firing pattern produced solely by the perforant path input and by the effect of the recurrent connections, we have been able to show (Treves and Rolls, 1992) that an input of the perforant path type, alone, is unable to direct efficient information storage. Such an input is too weak, it turns out, to drive the firing of the cells, as the “dynamics” of the network is dominated by the randomizing effect of the RCs. On the other hand, an autoassociative memory network needs afferent inputs to apply the retrieval cue to the network. We have shown (Treves and Rolls, 1992) that the perforant path system is likely to be the one involved in relaying the cues that initiate retrieval in CA3. The concept is that to initiate retrieval, a numerically large input (the perforant path system, see Figure 2) is useful so that even a partial cue is sufficient [see Equation 17 of Treves and Rolls (1992)]; and that the retrieval cue need not be very strong, as the RCs (in CA3) then take over in the retrieval process to produce good recall (Treves and Rolls, 1992, 2008). In this scenario, the perforant path to CA3 synapses operate as a pattern associator, the quantitative properties of which are described elsewhere (Rolls and Treves, 1990, 1998; Rolls, 2008). If an incomplete recall cue is provided to a pattern association network using distributed input representations, then most of the output pattern will be retrieved, and in this sense *pattern association networks generalize between similar retrieval patterns to produce the correct output firing* (Rolls, 2008, 2014a), and this generalization performed at the perforant path synapses to CA3 cells helps in the further completion produced by the RC CA3-CA3 autoassociation process.

In contrast, during storage, strong signals, in the order of mV for each synaptic connection, are provided by the mossy fiber inputs to dominate the RC activations, so that the new pattern of CA3 cell firing can be stored in the CA3 RC connections (Treves and Rolls, 1992; Rolls, 2008).

### The dilution of the CA3 recurrent collateral connectivity enhances memory storage capacity and pattern completion

Figure 2 shows that in the rat, there are approximately 300,000 CA3 neurons, but only 12,000 RC synapses per neuron. The dilution of the connectivity is thus 12,000 / 300,000 = 0.04. The connectivity is thus not complete, and complete connectivity in an autoassociation network would make it simple, for the connectivity between the neurons would then be symmetric (i.e., the connection strength from any one neuron to another is matched by a connection of the same strength in the opposite direction), and this guarantees energy minima for the basins of attraction that will be stable, and a memory capacity than can be calculated (Hopfield, 1982). We have shown how this attractor type of network can be extended to have similar properties with diluted connectivity, and also with sparse representations with graded firing rates (Rolls and Treves, 1990; Treves, 1990, 1991; Treves and Rolls, 1991; Webb et al., 2011; Rolls and Webb, 2012).

However, the question has recently been asked about whether there are any advantages to diluted autoassociation or attractor networks compared to fully connected attractor networks (Rolls, 2012a). One biological property that may be a limiting factor is the number of synaptic connections per neuron, which is 12,000 in the CA3-CA3 network just for the RCs (see Figure 2). The number may be higher in humans, allowing more memories to be stored in the hippocampus than order 12,000. I note that the storage of large number of memories may be facilitated in humans because the left and right hippocampus appear to be much less connected between the two hemispheres than in the rat, which effectively has a single hippocampus (Rolls, 2008). In humans, with effectively two separate CA3 networks, one on each side of the brain, the memory storage capacity may be doubled, as the capacity is set by the number of RCs per neuron in each attractor network (Equation 3). In humans, the right hippocampus may be devoted to episodic memories with spatial and visual components, whereas the left hippocampus may be devoted to memories with verbal / linguistic components, i.e., in which words may be part of the episode (e.g., who said what to whom and when) (Barkas et al., 2010; Bonelli et al., 2010; Sidhu et al., 2013).

The answer that has been suggested to why the connectivity of the CA3 autoassociation network is diluted (and why neocortical recurrent networks are also diluted), is that this may help to reduce the probability of having two or more synapses between any pair of randomly connected neurons within the network, which it has been shown greatly impairs the number of memories that can be stored in an attractor network, because of the distortion that this produces in the energy landscape (Rolls, 2012a). In more detail, the hypothesis proposed is that the diluted connectivity allows biological processes that set up synaptic connections between neurons to arrange for there to be only very rarely more than one synaptic connection between any pair of neurons. If probabilistically there were more than one connection between any two neurons, it was shown by simulation of an autoassociation attractor network that such connections would dominate the attractor states into which the network could enter and be stable, thus strongly reducing the memory capacity of the network (the number of memories that can be stored and correctly retrieved), below the normal large capacity for diluted connectivity. Diluted connectivity between neurons in the cortex thus has an important role in allowing high capacity of memory networks in the cortex, and helping to ensure that the critical capacity is not reached at which overloading occurs leading to an impairment in the ability to retrieve any memories from the network (Rolls, 2012a). The diluted connectivity is thus seen as an adaptation that simplifies the genetic specification of the wiring of the brain, by enabling just two attributes of the connectivity to be specified (e.g., from a CA3 to another CA3 neuron chosen at random to specify the CA3 to CA3 RC connectivity), rather than which particular neuron should connect to which other particular neuron (Rolls and Stringer, 2000; Rolls, 2012a). Consistent with this hypothesis, there are NMDA receptors with the genetic specification that they are NMDA receptors on neurons of a particular type, CA3 neurons (as shown by the evidence from CA3-specific vs. CA1-specific NMDA receptor knockouts) (Rondi-Reig et al., 2001; Nakazawa et al., 2002, 2003, 2004). A consequence is that the vector of output neuronal firing in the CA3 region, i.e., the number of CA3 neurons, is quite large (300,000 neurons in the rat). The large number of elements in this vector may have consequences for the noise in the system, as we will see below.

The role of dilution in the connectivity of the CA3 RC connectivity includes enabling this large number of separate memories to be recalled from any part of each memory, that is, in pattern completion (Rolls, 2012a).

The dilution of the CA3-CA3 RC connectivity at 0.04 may be greater dilution than that in a local neocortical area, which is in the order of 0.1 (Rolls, 2008, 2012a). This is consistent with the hypothesis that the storage capacity of the CA3 system is at a premium, and so the dilution is kept to a low value (i.e., great dilution), as then there is lower distortion of the basins of attraction and hence the memory capacity is maximized (Rolls, 2012a).

### Noise and stability produced by the diluted connectivity and the graded firing rates in the CA3-CA3 attractor network

Many processes in the brain are influenced by the noise or variability of neuronal spike firing (Faisal et al., 2008; Rolls and Deco, 2010; Deco et al., 2013). The action potentials are generated in a way that frequently approximates a Poisson process, in which the spikes for a given mean firing rate occur at times that are essentially random (apart from a small effect of the refractory period), with a coefficient of variation of the interspike interval distribution (CV) near 1.0 (Rolls and Deco, 2010). The sources of the noise include quantal transmitter release, and noise in ion channel openings (Faisal et al., 2008). The membrane potential is often held close to the firing threshold, and then small changes in the inputs and the noise in the neuronal operations cause spikes to be emitted at almost random times for a given mean firing rate. Spiking neuronal networks with balanced inhibition and excitation currents and associatively modified recurrent synaptic connections can be shown to possess a stable attractor state where neuron spiking is approximately Poisson too (Amit and Brunel, 1997; Miller and Wang, 2006). The noise caused by the variability of individual neuron spiking which then affects other neurons in the network can play an important role in the function of such recurrent attractor networks, by causing for example an otherwise stable network to jump into a decision state (Wang, 2002; Deco and Rolls, 2006; Rolls and Deco, 2010). Attractor networks with this type of spiking-related noise are used in the brain for memory recall, and for decision-making, which in terms of the neural mechanism are effectively the same process (Rolls, 2008). Noise in attractor networks is useful for memory and decision-making, for it makes them non-deterministic, and this contributes to new solutions to problems, and indeed to creativity (Rolls and Deco, 2010; Rolls, 2014a).

To investigate the extent to which the diluted connectivity affects the dynamics of attractor networks in the cerebral cortex (which includes the hippocampus), we simulated an integrate-and-fire attractor network taking decisions between competing inputs with diluted connectivity of 0.25 or 0.1 but the same number of synaptic connections per neuron for the RC synapses within an attractor population as for full connectivity (Rolls and Webb, 2012). The results indicated that there was less spiking-related noise with the diluted connectivity in that the stability of the network when in the spontaneous state of firing increased, and the accuracy of the correct decisions increased. The decision times were a little slower with diluted than with complete connectivity. Given that the capacity of the network is set by the number of RC synaptic connections per neuron, on which there is a biological limit, the findings indicate that the stability of cortical networks, and the accuracy of their correct decisions or memory recall operations, can be increased by utilizing diluted connectivity and correspondingly increasing the number of neurons in the network (which may help to smooth the noise), with little impact on the speed of processing of the cortex. Thus, diluted connectivity can decrease cortical spiking-related noise, and thus enhance the reliability of memory recall, which includes completion from a partial recall cue (Rolls and Webb, 2012).

Representations in the neocortex and in the hippocampus are often distributed with graded firing rates in the neuronal populations (Rolls and Treves, 2011). The firing rate probability distribution of each neuron to a set of stimuli is often exponential or gamma (Rolls and Treves, 2011). These graded firing rate distributed representations are present in the hippocampus, both for place cells in rodents and for spatial view cells in the primate (O'Keefe, 1979; McNaughton et al., 1983; O'Keefe and Speakman, 1987; Rolls et al., 1997a, 1998; Robertson et al., 1998; Georges-François et al., 1999; Rolls, 2008; Rolls and Treves, 2011). In processes in the brain such as memory recall in the hippocampus or decision-making in the cortex that are influenced by the noise produced by the close to random spike timings of each neuron for a given mean rate, the noise with this graded type of representation may be larger than with the binary firing rate distribution that is usually investigated. In integrate-and-fire simulations of an attractor decision-making network, we showed that the noise is indeed greater for a given sparseness of the representation for graded, exponential, than for binary firing rate distributions (Webb et al., 2011). The greater noise was measured by faster escaping times from the spontaneous firing rate state when the decision cues are applied, and this corresponds to faster decision or reaction times. The greater noise was also evident as less stability of the spontaneous firing state before the decision cues are applied. The implication is that spiking-related noise will continue to be a factor that influences processes such as decision-making, signal detection, short-term memory, and memory recall and completion (including in the CA3 network) even with the quite large networks found in the cerebral cortex. In these networks there are several thousand RC synapses onto each neuron. The greater noise with graded firing rate distributions has the advantage that it can increase the speed of operation of cortical circuitry (Webb et al., 2011). The graded firing rates also by operating in a non-linear network effectively increase the sparseness of the representation, and this itself is a pattern separation effect (Webb et al., 2011).

## Pattern separation of CA3 cell populations encoding different memories

For the CA3 to operate with high capacity as an autoassociation or attractor memory, the sets of CA3 neurons that represent each event to be stored and later recalled need to be as uncorrelated from each other as possible. Correlations between patterns reduce the memory capacity of an autoassociation network (Marr, 1971; Kohonen, 1977, 1984; Kohonen et al., 1981; Rolls and Treves, 1998), and because storage capacity is at a premium in an episodic memory system, there are several mechanisms that reduce the correlations between the firing of the population vectors of CA3 neuron firing each one of which represents a different event to be stored in memory. In the theoretical physics approach to the capacity of attractor networks, it is indeed assumed that the different vectors of firing rates to be stored are well separated from each other, by drawing each vector of firing at random, and by assuming very large (infinite) numbers of neurons in each pattern (Hopfield, 1982; Rolls and Treves, 1998).

We have proposed that there are several mechanisms that help to achieve this pattern separation, namely the mossy fiber pattern separation effect produced by the small number of connections received by a CA3 neuron from mossy fibers which dominate the CA3 cell firing; the expansion recoding, and the sparse representation provided by the dentate granule cells that form the mossy fiber synapses; and the sparseness of the CA3 cell representation. Neurogenesis of dentate granule cells is a fifth potential contributor to achieving pattern separation of CA3 cell firing. The five factors are described next. Before this, it is remarked that some of this architecture may be special to the hippocampus, and not found in the neocortex, because of the importance of storing and retrieving large numbers of (episodic) memories in the hippocampus. The neocortex in contrast is more concerned with building new representations for which competitive learning is more important, and thus neocortical circuitry does not use a mossy fiber system to produce new random sets of neurons activated (Rolls, 2008).

### Pattern separation and the sparse connectivity of the mossy fibre inputs to CA3 cells

We hypothesize that the mossy fiber inputs force efficient information storage by virtue of their strong and sparse influence on the CA3 cell firing rates (Rolls, 1987, 1989b,c; Treves and Rolls, 1992). [The strong effects likely to be mediated by the mossy fibres were also emphasized by McNaughton and Morris (1987) and McNaughton and Nadel (1990)]. We (Rolls and Treves) (Rolls, 1987, 1989b,c, 1990b, 2008; Treves and Rolls, 1992; Rolls and Treves, 1998) hypothesize that the mossy fiber input appears to be particularly appropriate in several ways. First, the fact that mossy fiber synapses are large and located very close to the soma makes them relatively powerful in activating the postsynaptic cell. Second, the firing activity of dentate granule cells appears to be very sparse (Jung and McNaughton, 1993; Leutgeb et al., 2007) and this, together with the small number of connections on each CA3 cell, produces a sparse signal, which can then be transformed into sparse firing activity in CA3 by a threshold effect. The hypothesis is that the mossy fiber sparse connectivity solution performs the appropriate function to enable learning to operate correctly in CA3 (Treves and Rolls, 1992; Cerasti and Treves, 2010). The perforant path input would, the quantitative analysis shows, not produce a pattern of firing in CA3 that contains sufficient information for learning (Treves and Rolls, 1992) (see further section 3.2.6).

The particular property of the small number of mossy fiber connections onto a CA3 cell, approximately 46 (see Figure 2), is that this has a **randomizing effect** on the representations set up in CA3, so that they are as different as possible from each other (Rolls, 1989b,c, 2008; Treves and Rolls, 1992; Rolls and Treves, 1998; Rolls and Kesner, 2006). (This means for example that place cells in a given environment are well separated to cover the whole space.) The result is that any one event or episode will set up a representation that is very different from other events or episodes, because the set of CA3 neurons activated for each event is random. This is then the optimal situation for the CA3 RC effect to operate, for it can then associate together the random set of neurons that are active for a particular event (for example an object in a particular place), and later recall the whole set from any part. It is because the representations in CA3 are unstructured, or random, in this way that large numbers of memories can be stored in the CA3 autoassociation system, and that interference between the different memories is kept as low as possible, in that they are maximally different from each other (Hopfield, 1982; Treves and Rolls, 1991; Rolls and Treves, 1998; Rolls, 2008).

The requirement for a small number of mossy fiber connections onto each CA3 neuron applies not only to discrete (Treves and Rolls, 1992) but also to spatial representations, and some learning in these connections, whether associative or not, can help to select out the small number of mossy fibres that may be active at any one time to select a set of random neurons in the CA3 (Cerasti and Treves, 2010). Any learning may help by reducing the accuracy required for a particular number of mossy fiber connections to be specified genetically onto each CA3 neuron. The optimal number of mossy fibres for the best information transfer from dentate granule cells to CA3 cells is in the order of 35–50 (Treves and Rolls, 1992; Cerasti and Treves, 2010). The mossy fibres also make connections useful for feed forward inhibition in CA3 (Acsady et al., 1998), which is likely to be useful to help in the sparse representations being formed in CA3.

On the basis of these and other points, we predicted that the mossy fibres may be necessary for new learning in the hippocampus, but may not be necessary for the recall of existing memories from the hippocampus (Treves and Rolls, 1992; Rolls and Treves, 1998; Rolls, 2008). Experimental evidence consistent with this prediction about the role of the mossy fibres in learning has been found in rats with disruption of the dentate granule cells (Lassalle et al., 2000) (section Pattern Separation Performed by Dentate Granule cells).

We (Rolls and Kesner, 2006) have hypothesized that non-associative plasticity of mossy fibres (see Brown et al., 1989, 1990) might have a useful effect in enhancing the signal-to-noise ratio, in that a consistently firing mossy fiber would produce non-linearly amplified currents in the postsynaptic cell, which would not happen with an occasionally firing fiber (Treves and Rolls, 1992). This plasticity, and also learning in the dentate, would also have the effect that similar fragments of each episode (e.g., the same environmental location) recurring on subsequent occasions would be more likely to activate the same population of CA3 cells, which would have potential advantages in terms of economy of use of the CA3 cells in different memories, and in making some link between different episodic memories with a common feature, such as the same location in space. Consistent with this, dentate neurons that fire repeatedly are more effective in activating CA3 neurons (Henze et al., 2002).

As acetylcholine turns down the efficacy of the RC synapses between CA3 neurons (Hasselmo et al., 1995; Giocomo and Hasselmo, 2007), then cholinergic activation also might help to allow external inputs from the mossy fibers rather than the internal RC inputs to dominate the firing of the CA3 neurons during learning, as the current theory proposes. If cholinergic activation at the same time facilitated LTP in the RCs (as it appears to in the neocortex), then cholinergic activation could have a useful double role in facilitating new learning at times of behavioral activation (Hasselmo et al., 1995; Giocomo and Hasselmo, 2007), when presumably it may be particularly relevant to allocate some of the limited memory capacity to new memories.

### Pattern separation and the sparseness of the firing of the dentate granule cell input via the mossy fibres to CA3 cells

The firing activity of dentate granule cells appears to be very sparse (Jung and McNaughton, 1993; Leutgeb et al., 2007) and this, together with the small number of dentate mossy fiber connections on each CA3 cell, produces a sparse signal, which can then be transformed into sparse firing activity in CA3 by a threshold effect. The pattern separation mechanisms that enable the dentate to provide a sparse firing input to CA3 are described below.

### Pattern separation and the large number of dentate granule cells providing inputs via the mossy fibres to CA3 cells

Expansion recoding can decorrelate input patterns, and this can be performed by a stage of competitive learning with a large number of neurons (Rolls, 2008). A mechanism like this appears to be performed by the dentate granule cells, which are numerous (1 × 10^6^ in the rat, compared to 300,000 CA3 cells), have associatively modifiable synapses (required for a competitive network), and strong inhibition provided by the inhibitory interneurons. This may not represent expansion of numbers relative to the number of entorhinal cortex cells, but the principle of a large number of dentate granule cells, with competitive learning and strong inhibition through inhibitory interneurons, would produce a decorrelation of signals like that achieved by expansion recoding (Rolls, 2008).

### Sparseness of the CA3 cell representation and pattern separation

The firing of CA3 cells is relatively sparse, and this helps to decorrelate different population vectors of CA3 cell firing for different memories. [Sparse representations are more likely to be decorrelated with each other (Rolls, 2008).] Evidence on the sparseness of the CA3 cell representation in rats includes evidence that CA3 cell ensembles may support the fast acquisition of detailed memories by providing a locally continuous, but globally orthogonal spatial representation, onto which new sensory inputs can rapidly be associated (Leutgeb and Leutgeb, 2007). In the macaque hippocampus, in which spatial view cells are found (Rolls et al., 1997a, 1998; Robertson et al., 1998; Georges-François et al., 1999), for the representation of 64 locations around the walls of the room, the mean single cell sparseness *a*^s^ was 0.34, and the mean population sparseness *a*^p^ was 0.33 (Rolls et al., 1998; Rolls, 2008; Rolls and Treves, 2011). For comparison, the corresponding values for inferior temporal cortex neurons tuned to objects and faces were 0.77 (Franco et al., 2007; Rolls, 2008; Rolls and Treves, 2011); for taste and oral texture neurons in the insular cortex the population sparseness was 0.71; for taste and oral texture neurons in the orbitofrontal cortex was 0.61; and for taste and oral texture neurons in the amygdala was 0.81 (Rolls, 2008; Rolls and Treves, 2011). Thus, the evidence is that the hippocampal CA3 / pyramidal cell representation is more sparse in macaques than in neocortical areas and the amygdala, and this is consistent with the importance in hippocampal CA3 of using a sparse representation to produce a large memory capacity.

### Neurogenesis of dentate granule cells to provide new representations in CA3 uncorrelated with previous CA3 representations

If adult neurogenesis in the dentate gyrus does prove to be functionally relevant, its computational role could be to facilitate pattern separation for new patterns, by providing new dentate granule cells with new sets of random connections to CA3 neurons (Rolls, 2010b). Consistent with the dentate spatial pattern separation hypothesis (Rolls, 1989b,c, 1996b, 2008; Treves and Rolls, 1992, 1994), in mice with impaired dentate neurogenesis, spatial learning in a delayed non-matching-to-place task in the radial arm maze was impaired for arms that were presented with little separation, but no deficit was observed when the arms were presented farther apart (Clelland et al., 2009). Consistently, impaired neurogenesis in the dentate also produced a deficit for small spatial separations in an associative object-in-place task (Clelland et al., 2009).

### The direct perforant path to CA3 cell input: poor at pattern separation and forcing a new memory pattern into CA3 cell firing

It has been suggested that the feed forward connectivity from the entorhinal cortex via the perforant path to the CA3 neurons may act as a feed forward pattern association network that is more important than the CA3-CA3 RC autoassociation system (Cheng, 2013). The quantitative properties of pattern association networks are described elsewhere (Rolls and Treves, 1990, 1998; Rolls, 2008). If an incomplete recall cue is provided to a pattern association network using distributed input representations, then most of the output pattern will be retrieved, and in this sense *pattern association networks do generalize*. (As noted above, pattern association networks do not perform pattern completion, in that the unconditioned stimulus cannot recall the conditioned stimulus.) The analyses described in these sources shows that the capacity of pattern association networks (the maximum number of memories that can be stored and retrieved, here denoted by *p*_max_) is approximately

(4) $$p_{\max}\approx \frac{C^{\text{PA}}}{\left[a_{\text{o}}\ln(1/a_{\text{o}})\right]}$$

where *C*^PA^ is the number of feed forward associatively modifiable connections per neuron, and *a*_*o*_ is the sparseness of the representation in the output neurons of the pattern associator (Rolls, 2008). Given that there are fewer feed forward (perforant path) synaptic connections onto CA3 neurons (3,600) than recurrent synaptic connections between CA3 neurons (12,000 in the rat) (see Figure 2), then the capacity of the feed forward system would be considerably smaller than that of the RC CA3-CA3 system. [It is noted that the *a*_o_ of Equation (4) would be the same number as the *a* of equation (3), as that is just the sparseness of the firing of the population of CA3 neurons. The number of perforant path synapses is sufficiently large that it can generalize given even a partial retrieval pattern, so that the CA3-CA3 connections can then complete the retrieval, given that the recall signal for the perforant path pattern associator is proportional to the square root of the number of perforant path synapses, as shown by Equation 17 of Treves and Rolls (1992).] The feed forward hypothesis (Cheng, 2013) thus has a strong argument against it of storage capacity, which would be much less (~3600 / 12,000) than that of the CA3-CA3 RC system operating as an autoassociation memory. Another disadvantage of the feed forward hypothesis is that the attractor properties of the CA3-CA3 connections would be lost, and these potentially contribute to holding one or more items simultaneously active in short-term memory (Rolls, 2008; Rolls et al., 2013), and providing a basis for temporal order memory as described in section The Dilution of the CA3 Recurrent Collateral Connectivity Enhances Memory Storage Capacity and Pattern Completion. Another disadvantage is that we have been able to show (Treves and Rolls, 1992) that an input of the perforant path type, alone, is unable to direct efficient information storage. Such an input is too weak, it turns out, to drive the firing of the cells, as the “dynamics” of the network is dominated by the randomizing effect of the RCs. Another disadvantage of the feed forward hypothesis is that a pattern associator may not with an incomplete cue be able to recall the complete pattern that was stored, whereas an attractor network has the property that it can fall into an attractor basin that can reflect perfect retrieval of the complete memory (Rolls and Treves, 1998; Rolls, 2008).

## Pattern separation performed by dentate granule cells

The theory is that the dentate granule cell stage of hippocampal processing which precedes the CA3 stage acts as a competitive network in a number of ways to produce during learning the sparse yet efficient (i.e., non-redundant) representation in CA3 neurons that is required for the autoassociation implemented by CA3 to perform well (Rolls, 1989b,c, 1990b; Treves and Rolls, 1992; Rolls and Kesner, 2006; Rolls et al., 2006). An important property for episodic memory is that the dentate by acting in this way would perform pattern separation (or orthogonalization) (Rolls, 1989b; Treves and Rolls, 1992; Rolls and Kesner, 2006; Rolls et al., 2006), enabling the hippocampus to store different memories of even similar events, and this prediction has been confirmed (Gilbert et al., 2001; Rolls and Kesner, 2006; Leutgeb and Leutgeb, 2007; McHugh et al., 2007; Goodrich-Hunsaker et al., 2008; Rolls, 2008; Kesner et al., 2012). Consistently with this evidence for pattern separation by dentate granule cells, in rats small changes in the shape of the environment in which rats are exploring can substantially alter the activity patterns among place-modulated granule cells (Leutgeb et al., 2007).

As just described, the dentate granule cells could be important in helping to build and prepare spatial representations for the CA3 network. The actual representation of space in the primate hippocampus includes a representation of spatial view (Rolls et al., 1997a, 1998; Robertson et al., 1998; Georges-François et al., 1999; Rolls and Xiang, 2006), whereas in the rat hippocampus it is of the place where the rat is. The representation in the rat may be related to the fact that with a much less developed visual system than the primate, the rat's representation of space may be defined more by the olfactory and tactile as well as distant visual cues present, and may thus tend to reflect the place where the rat is. However, the spatial representations in the rat and primate could arise from essentially the same computational process as follows (Rolls, 1999; De Araujo et al., 2001). The starting assumption is that in both the rat and the primate, the dentate granule cells (and the CA3 and CA1 pyramidal cells) respond to combinations of the inputs received. In the case of the primate, a combination of visual features in the environment will, because of the fovea providing high spatial resolution over a typical viewing angle of perhaps 10–20 degrees, result in the formation of a spatial view cell, the effective trigger for which will thus be a combination of visual features within a relatively small part of space. In contrast, in the rat, given the very extensive visual field subtended by the rodent retina, which may extend over 180-270 degrees, a combination of visual features formed over such a wide visual angle would effectively define a position in space that is a place (De Araujo et al., 2001).

**Figure 4:**
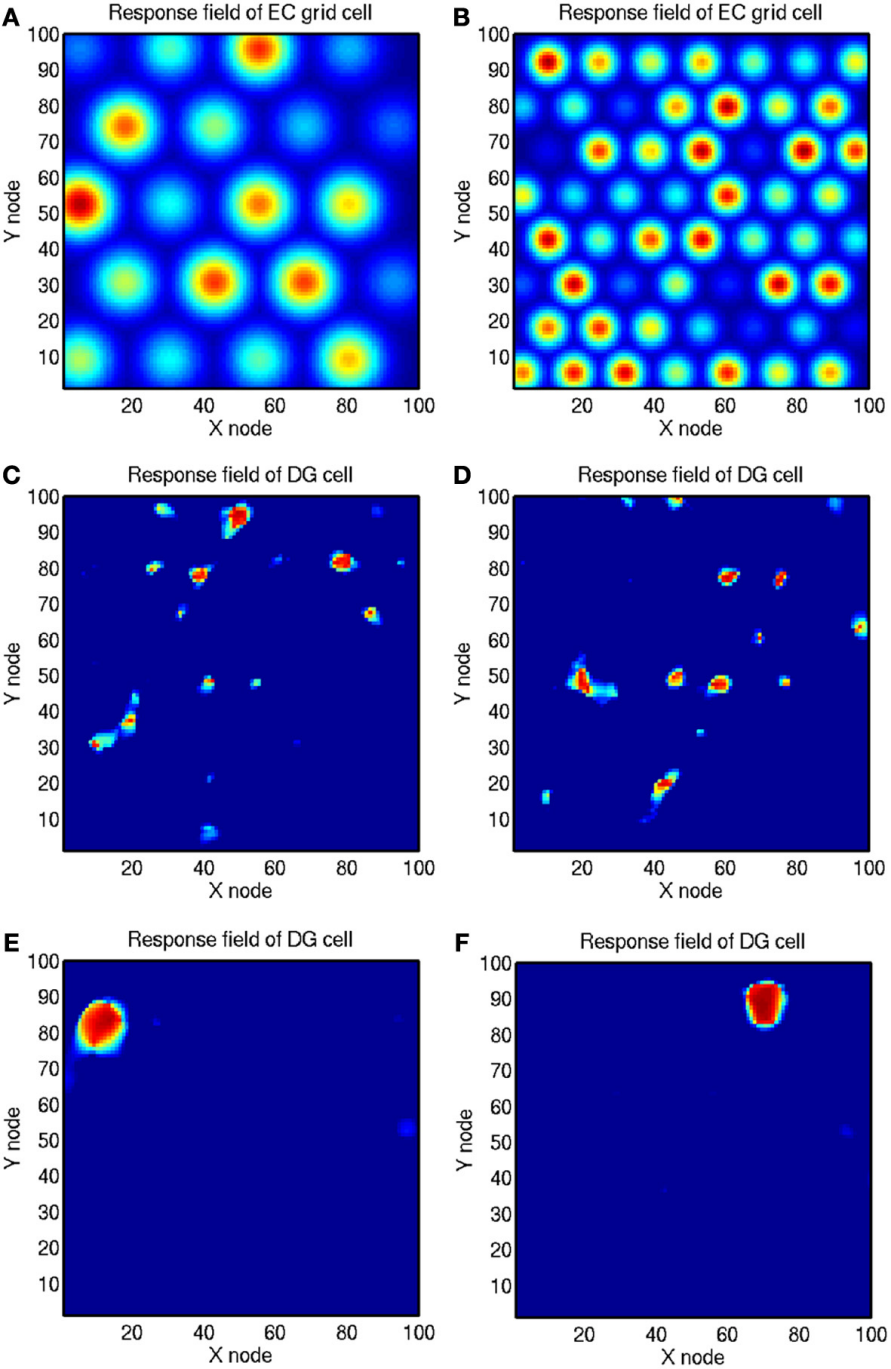
**Simulation of competitive learning in the dentate gyrus to produce place cells from the entorhinal cortex grid cell inputs. (A** and **B)** Firing rate profiles of two entorhinal cortex (EC) grid cells with frequencies of 4 and 7 cycles. **(C** and **D)** Firing rate profiles of two dentate gyrus (DG) cells with no training using competitive learning. **(E** and **F)** Firing rate profiles of two dentate gyrus (DG) cells trained using competitive learning. (After Rolls et al., 2006).

The entorhinal cortex contains grid cells, which have high firing in the rat in a two-dimensional spatial grid as a rat traverses an environment, with larger grid spacings in the ventral entorhinal cortex (Fyhn et al., 2004; Hafting et al., 2005). This may be a system optimized for path integration (McNaughton et al., 2006) which may self-organize during locomotion with longer time constants producing more widely spaced grids in the ventral entorhinal cortex (Kropff and Treves, 2008). How are the grid cell representations, which would not be suitable for association of an object or reward with a place to form an episodic memory, transformed into a place representation that would be appropriate for this type of episodic memory? I have proposed that this could be implemented by a competitive network (Rolls, 2008) in the dentate gyrus which operates to form place cells, implemented by each dentate granule cell learning to respond to particular combinations of entorhinal cortex cells firing, where each combination effectively specifies a place, and this has been shown to be feasible computationally (Rolls et al., 2006). The sparse representations in the dentate gyrus, implemented by the mutual inhibition through inhibitory interneurons and competitive learning, help to implement this “pattern separation” effect (Rolls, 1989b,c; Rolls and Treves, 1998; Rolls, 2008). The investigations showed that learning in the perforant path to dentate granule cell representation, and the sparse representation in the dentate granule cells, are both important in the formation of place-like fields in dentate granule cells from the grid cells in the entorhinal cortex (Rolls et al., 1997a, 1998; Robertson et al., 1998; Georges-François et al., 1999). To illustrate this, Figure 4 shows from these simulations the responses of the simulated grid cells (**A,B**), the dentate receptive fields formed by feed forward connections and a sparse representation in the dentate gyrus (**C,D**), and the dentate receptive fields formed when Hebbian synaptic modification and training is included in the feed forward connections to implement competitive learning (**E,F**). It is only with the full competitive learning that the dentate receptive fields self-organized to become small place-like receptive fields (Rolls et al., 2006) similar to those found in the rat dentate granule cells.

In primates, there is now evidence that there is a grid-cell like representation in the entorhinal cortex, with neurons having grid-like firing as the monkey moves the eyes across a spatial scene (Killian et al., 2012). Similar competitive learning processes may transform these entorhinal cortex “spatial view grid cells” into hippocampal spatial view cells, and may help with the idiothetic (produced in this case by movements of the eyes) update of spatial view cells (Robertson et al., 1998). The presence of spatial view grid cells in the entorhinal cortex of primates (Killian et al., 2012) is of course predicted from the presence of spatial view cells in the primate CA3 and CA1 regions (Rolls et al., 1997a, 1998; Robertson et al., 1998; Georges-François et al., 1999; Rolls and Xiang, 2006; Rolls, 2008). Further support for this type of representation of space being viewed “out there” rather than where one is located as for rat place cells is that cells in the human entorhinal cortex with spatial view grid-like properties have now been described (Jacobs et al., 2013).

## CA1 cells and pattern completion prior to hippocampo-directed recall to the neocortex

The CA3 cells connect to the CA1 cells by the Schaeffer collateral synapses. The associative modifiability in this connection helps the full information present in CA3 to be retrieved in the CA1 neurons (Treves and Rolls, 1994; Rolls, 1995; Treves, 1995; Schultz and Rolls, 1999). Part of the hypothesis is that the separate sub-parts of an episodic memory, which must be represented separately in CA3 to allow for completion, can be combined together by competitive learning in CA1 to produce an efficient retrieval cue for the recall via the backprojection pathways to the neocortex of memories stored in the neocortex (Rolls, 1989a,b, 1995, 1996b; Treves and Rolls, 1994). Associative recall by pattern association in the CA3 to CA1 feed forward connections is a prominent property which implements generalization, so that if completion in CA3 was not perfect, CA1 could generalize to the closest CA1 representation (Rolls, 1995, 2008; Schultz et al., 2000).

## Backprojections to the neocortex, and memory retrieval from the hippocampus to provide a complete neocortical memory representation

The need for information to be retrieved from the hippocampus to affect other brain areas was noted in the Introduction. The way in which this could be implemented via backprojections to the neocortex (Treves and Rolls, 1994; Rolls, 1995, 1996b, 2008, 2010b) is considered here in the context of recalling a complete memory representation in the complete set of cortical areas that provide inputs to the hippocampus (see Figure 1).

It is suggested that the modifiable connections from the CA3 neurons to the CA1 neurons allow the whole episode in CA3 to be produced in CA1. The CA1 neurons would then activate, via their termination in the deep layers of the entorhinal cortex, at least the pyramidal cells in the deep layers of the entorhinal cortex (see Figure 1). These entorhinal cortex layer 5 neurons would then, by virtue of their backprojections (Lavenex and Amaral, 2000; Witter et al., 2000a) to the parts of cerebral cortex that originally provided the inputs to the hippocampus, terminate in the superficial layers (including layer 1) of those neocortical areas, where synapses would be made onto the distal parts of the dendrites of the (superficial and deep) cortical pyramidal cells (Rolls, 1989a,b,c; Markov et al., 2013). The areas of cerebral neocortex in which this recall would be produced could include multimodal cortical areas [e.g., the cortex in the superior temporal sulcus which receives inputs from temporal, parietal and occipital cortical areas, and from which it is thought that cortical areas such as 39 and 40 related to language developed; and the orbitofrontal and anterior cingulate cortex to retrieve the reward / affective aspects of an episodic memory (Rolls, 2014a,b)], and also areas of unimodal association cortex (e.g., inferior temporal visual cortex). The backprojections, by recalling previous episodic events, could provide information useful to the neocortex in the building of new representations in the multimodal and unimodal association cortical areas, which by building new long-term and structured representations can be considered as a form of memory consolidation (Rolls, 1989a,b,c, 1990a,b, 2008), or in organizing actions.

The hypothesis of the architecture with which this would be achieved is shown in Figure 1. The feed forward connections from association areas of the cerebral neocortex (solid lines in Figure 1), show major convergence as information is passed to CA3, with the CA3 autoassociation network having the smallest number of neurons at any stage of the processing. The backprojections allow for divergence back to neocortical areas. The way in which I suggest that the backprojection synapses are set up to have the appropriate strengths for recall is as follows (Rolls, 1989a,b,c). During the setting up of a new episodic memory, there would be strong feed forward activity progressing toward the hippocampus. During the episode, the CA3 synapses would be modified, and via the CA1 neurons and the subiculum, a pattern of activity would be produced on the backprojecting synapses to the entorhinal cortex. Here the backprojecting synapses from active backprojection axons onto pyramidal cells being activated by the forward inputs to entorhinal cortex would be associatively modified. A similar process would be implemented at preceding stages of neocortex, that is in the parahippocampal gyrus/perirhinal cortex stage, and in association cortical areas, as shown in Figure 1.

The concept is that during the learning of an episodic memory, cortical pyramidal cells in at least one of the stages would be driven by forward inputs, but would simultaneously be receiving backprojected activity (indirectly) from the hippocampus which would by pattern association from the backprojecting synapses to the cortical pyramidal cells become associated with whichever cortical cells were being made to fire by the forward inputs. Then later on, during recall, a recall cue from perhaps another part of cortex might reach CA3, where the firing during the original episode would be completed. The resulting backprojecting activity would then, as a result of the pattern association learned previously, bring back the firing in any cortical area that was present during the original episode. Thus, retrieval involves reinstating the activity that was present in different cortical areas that was present during the learning of an episode. (The pattern association is also called heteroassociation, to contrast it with autoassociation. The pattern association operates at multiple stages in the backprojection pathway, as made evident in Figure 1). If the recall cue was an object, this might result in recall of the neocortical firing that represented the place in which that object had been seen previously. As noted elsewhere in this paper and by McClelland et al. (1995), that recall might be useful to the neocortex to help it build new semantic memories, which might inherently be a slow process and is not part of the theory of recall.

A plausible requirement for a successful hippocampo-directed recall operation, is that the signal generated from the hippocampally retrieved pattern of activity, and carried backwards toward neocortex, remain undegraded when compared to the noise due, at each stage, to the interference effects caused by the concurrent storage of other patterns of activity on the same backprojecting synaptic systems. That requirement is equivalent to that used in deriving the storage capacity of such a series of heteroassociative memories, and it was shown by Treves and Rolls (1994, 1991) that the maximum number of independently generated activity patterns that can be retrieved is given, essentially, by the same formula as (3) above where, however, *a* is now the sparseness of the representation at any given stage, and *C* is the average number of (back-)projections each cell of that stage receives from cells of the previous one. (*k*' is a similar slowly varying factor to that introduced above.) If *p* is equal to the number of memories held in the hippocampal memory, it is limited by the retrieval capacity of the CA3 network, *p*_max_. Putting together the formula for the latter with that shown here, one concludes that, roughly, the requirement implies that the number of afferents of (indirect) hippocampal origin to a given neocortical stage (*C*^HBP^), must be *C*^HBP^ = C^RC^*a*_nc_/*a*_CA3_, where *C*^RC^ is the number of RCs to any given cell in CA3, the average sparseness of a representation is *a*_nc_, and *a*_CA3_ is the sparseness of memory representations there in CA3.

The above requirement is very strong: even if representations were to remain as sparse as they are in CA3, which is unlikely, to avoid degrading the signal, *C*^HBP^ should be as large as *C*^RC^, i.e., 12,000 in the rat. If then *C*^HBP^ has to be of the same order as *C*^RC^, one is led to a very definite conclusion: a mechanism of the type envisaged here could not possibly rely on a set of monosynaptic CA3-to-neocortex backprojections. This would imply that, to make a sufficient number of synapses on each of the vast number of neocortical cells, each cell in CA3 has to generate a disproportionate number of synapses (i.e., *C*^HBP^ times the ratio between the number of neocortical and that of CA3 cells). The required divergence can be kept within reasonable limits only by assuming that the backprojecting system is polysynaptic, provided that the number of cells involved grows gradually at each stage, from CA3 back to neocortical association areas (Treves and Rolls, 1994) (cf. Figure 1).

The theory of recall by the backprojections thus provides a quantitative account of why the cerebral cortex has as many backprojection as forward projection connections (Rolls, 2008).

These concepts show how the backprojection system to neocortex can be conceptualized in terms of pattern completion, as follows. The information that is present when a memory is formed may be present in different areas of the cerebral cortex, for example of a face in a temporal cortex face area (Rolls, 2012b), of a spatial location in a neocortical location area, and of a reward received in the orbitofrontal cortex (Rolls, 2014a). To achieve detailed retrieval of the memory, reinstatement of the activity during recall of the neuronal activity during the original memory formation may be needed. This is what the backprojection system described could achieve, and is a form of completion of the information that was represented in the different cortical areas when the memory as formed. In particular, the concept of completion here is that if a recall cue from a visual object area is provided, then the emotional parts of the episodic memory can be recalled in the orbitofrontal cortex, and the spatial parts in parietal cortical areas, with the result that a complete memory is retrieved, with activity recalled into several higher-order cortical areas. Because such a wide set of different neocortical areas must be content-addressed, a multistage feedback system is required, to keep the number of synapses per neuron in the backprojection pathways down to reasonable numbers. (Having CA1 directly address neocortical areas would require each CA1 neuron to have tens of millions of synapses with cortical neurons. That is part of the computational problem solved by the multistage backprojection system shown in Figure 1.) Thus, the backprojection system with its series of pattern associators can each be thought of as retrieving the complete pattern of cortical activity in many different higher-order cortical areas that was present during the original formation of the episodic memory.

Further aspects of the operation of the backprojecting systems are described elsewhere (Rolls, 2008).

## Tests of pattern separation and pattern completion

There is now a large literature on tests of pattern separation and pattern completion in the hippocampus (Nakazawa et al., 2002, 2003; Wills et al., 2005; Rolls and Kesner, 2006; Kesner, 2007, 2013; Leutgeb et al., 2007; McHugh et al., 2007; Hunsaker and Kesner, 2008; Giocomo et al., 2011; Jezek et al., 2011; Kesner et al., 2012; Nakashiba et al., 2012; Hunsaker and Kesner, 2013), and a brief summary of some of the findings is provided next. An important point is that the theory (Rolls, 1987, 1989a,b,c, 1990b,a, 1991, 1995, 1996b, 2008, 2010b; Treves and Rolls, 1991, 1992, 1994; Rolls et al., 2013; Rolls and Treves, 1998; Rolls and Kesner, 2006; Rolls and Deco, 2010) is a quantitative theory of hippocampal function, and addresses how pattern separation and pattern completion are important in enabling the hippocampal system to operate up to capacity, which is in the order of tens of thousands of different memories. Some predictions from the theory may only hold when the system is well loaded, that is tested when the system is operating with thousands of memories, for then the pattern separation will be important. It is possible to test the predictions in simulations, where the system can be trained up to capacity (Rolls, 1995, 2012a; Rolls et al., 1997b). *In vivo*, it may be useful to test the storage and recall of as many memories as possible, and in addition testing animals kept in environments where memories of the hippocampal type are needed may also help to test hypotheses in situations where the hippocampus has been at least moderately well loaded with many different memories.

### Dentate granule cells

The theory predicts that pattern separation is performed by competitive learning by the dentate granule cells. Evidence consistent with this has been found neurophysiologically in the small sparsely encoded place fields of dentate neurons (Jung and McNaughton, 1993; Leutgeb and Leutgeb, 2007) and their reflection in CA3 neurons (Leutgeb and Leutgeb, 2007). Further, and consistent with the theory, it has been shown that selective dentate lesions in rats (Gilbert et al., 2001; Gilbert and Kesner, 2003; Rolls and Kesner, 2006; Goodrich-Hunsaker et al., 2008; Rolls, 2008; Hunsaker and Kesner, 2013; Kesner, 2013) or dentate granule cell NMDA receptor knockouts in mice (McHugh et al., 2007) impair spatial, object-place (or reward-place: remembering where to find a reward) association tasks especially when the places are close together and require pattern separation before storage in CA3.

### Mossy fiber inputs to CA3 and learning

The theory predicts that the dentate granule cell mossy fiber system of inputs to the CA3 neurons is necessary to store spatial memories, but not to recall them. Lassalle et al. (2000) have obtained evidence consistent with this in rats with damage to the mossy fiber system (Lassalle et al., 2000), and there is further evidence consistent with this (Lee and Kesner, 2004; Rolls and Kesner, 2006; Daumas et al., 2009).

### Perforant path inputs to CA3 and recall

The theory predicts that the direct perforant path input from the entorhinal cortex to the CA3 cells (which bypasses the dentate granule cells) is involved in the recall of memory from the CA3 system, and Lee and Kesner (2004) have obtained evidence consistent with this in a Hebb-Williams maze recall task.

### CA3 and pattern completion

The theory predicts that the CA3 system is especially important in object-place or reward-place tasks in which associations must be formed between any spatial location and any object (referred to as **arbitrary associations**). There is much evidence from subregion analyses involving disruption of CA3 that CA3 is necessary for arbitrary associations between places and objects or rewards (Gilbert and Kesner, 2003; Rolls and Kesner, 2006; Hunsaker and Kesner, 2013). Similar impairments were obtained following deletion of CA3 NMDA receptors in mice in the acquisition of an odor-context paired associate learning task (Rajji et al., 2006). If place or time is not a component, associative tasks such as odor-object association are not impaired (Rolls and Kesner, 2006), underlining the fact that the hippocampus is especially involved in episodic types of associative memory which typically involve place and/or time.

The theory predicts that the CA3 is especially important in object-place or reward-place **completion** tasks, in which associations must be completed from a part of the whole. It has been shown that if completion from an incomplete cue is needed, then CA3 NMDA receptors are necessary (presumably to ensure satisfactory CA3-CA3 learning) even in a reference memory task (Nakazawa et al., 2002; Gold and Kesner, 2005; Hunsaker and Kesner, 2013).

The theory predicts that the CA3 system is especially needed in **rapid, one-trial object-place, learning and recall**. It has been shown that hippocampal NMDA receptors (necessary for Long Term Potentiation to occur) are needed for one-trial flavor-place association learning, and that hippocampal AMPA/kainate receptors are sufficient for the recall, though the hippocampal subregion involved was not tested (Day et al., 2003). In subregion studies, Kesner and colleagues have shown that CA3 lesions produce chance performance on a one-trial object-place recall task (Kesner et al., 2008) and other object-spatial tasks (Kesner and Rolls, 2001; Rolls and Kesner, 2006). For example, CA3 lesions produced chance performance on both a one-trial object-place recall and place-object recall task (Kesner et al., 2008). This is evidence that CA3 supports arbitrary associations as well as episodic memory based on 1-trial learning. A control fixed visual conditional to place task with the same delay was not impaired, showing that it is recall after one-trial (or rapid, episodic) learning that is impaired (Kesner et al., 2008). CA3 NMDA receptors are as predicted by the theory necessary for rapid / one-trial spatial learning, as shown by a mouse knockout study by Nakazawa, Tonegawa and colleagues (Nakazawa et al., 2003, 2004; Tonegawa et al., 2003). We have shown that hippocampal CA3 neurons reflect the computational processes necessary for one-trial object-place event memory, used as a model for episodic memory (Rolls and Xiang, 2006).

Another type of test of the autoassociation (or attractor) hypothesis for CA3 has been to train rats in different environments, e.g., a square and a circular environment, and then test the prediction of the hypothesis that when presented with an environment ambiguous between these, that hippocampoal neurons will fall in an attractor state that represents one of the two previously learned environments, but not a mixture of the two environments. Evidence consistent with the hypothesis has been found (Wills et al., 2005). In a particularly dramatic example, it has been found that within each theta cycle, hippocampal pyramidal neurons may represent one or other of the learned environments (Jezek et al., 2011). This is an indication, predicted by Rolls and Treves (1998), that autoassociative memory recall can take place sufficiently rapidly to be complete within one theta cycle (120 ms), and that theta cycles could provide a mechanism for a fresh retrieval process to occur after a reset caused by the inhibitory part of each theta cycle, so that the memory can be updated rapidly to reflect a continuously changing environment, and not remain too long in an attractor state.

Evidence that the firing of hippocampal pyramidal cells in macaques is more sparse than in neocortical areas is described in section Sparseness of the CA3 Cell Representation and Pattern Separation. This is consistent with the premium placed in the hippocampus for storing and retrieving large numbers of independent memories.

The theory predicts that if primates including humans can form an episodic memory in which objects or people are seen at particular locations even though the observer viewing the space has never been to those locations “out there” in space, there should be a neural system in CA3 that can support such associations between places “out there” in a scene and objects. Exactly this is provided by the spatial view neurons Rolls and colleagues have discovered that are present in CA3 (Rolls et al., 1997a, 1998, 2005; Robertson et al., 1998; Georges-François et al., 1999; Rolls and Xiang, 2005, 2006). Place cells will not do for this type of episodic memory (Rolls, 2010b, 2013).

### Recall via CA1 to neocortex: a reverse hierarchy of pattern associators each performing pattern completion

The theory shows quantitatively, analytically, how memories could be retrieved from the hippocampus to the neocortex (Treves and Rolls, 1994), and this has been shown by simulation of the multistage hippocampal system including the entorhinal cortex, dentate, CA3, CA1, and return to the entorhinal cortex to recall the memory to be quantitatively realistic (Rolls, 1995).

It has been shown that after learning in hippocampal-dependent tasks, neocortical representations may change (Schwindel and McNaughton, 2011). Although this has been interpreted as the transfer of memories from the hippocampus to the neocortex (Schwindel and McNaughton, 2011), it should be noted that if the hippocampal representation changes as a result of learning, then the altered representation in CA1 will, even with fixed synaptic connections back to neocortex, alter neocortical firing, with no learning or actual ‘transfer’ involved. (This occurs whenever one vector of neuronal firing changes and influences another vector of neuronal firing through fixed connections.)

It has also been suggested that the transfer of information from the hippocampus to the neocortex occurs especially during sleep (Marr, 1971; Schwindel and McNaughton, 2011). My own view is that during waking would be the best time to retrieve a memory from the hippocampus to the neocortex by using the hippocampus to retrieve the complete episodic memory from a fragment. The retrieval would reinstate the neocortical activity present when the event was originally learned. The retrieved information now present in the neocortex could then be used to build new semantic memories, for example a narrative account of all the events that took place on one's fifth birthday party. During waking the building of semantic representations could be guided and organized by rational thought into useful semantic representations. To do this during sleep would run the risk of forming bizarre semantic representations of the type that we dream about during the unguided noise-driven stochastic firing during sleep (Rolls, 2008; Rolls and Deco, 2010). Further, the active recall during waking of memories from the hippocampus means that mainly relevant or useful memories would be retrieved from the hippocampus (not useless memories such as where one parked one's bicycle two weeks ago), and only these memories would tend to become incorporated into useful long-term semantic representations, allowing memories not retrieved from the hippocampus to be overwritten by new memories in the process of forgetting that involves using CA3 sets of neurons chosen at random for new episodic memories (Rolls, 2008).

Many further tests of the theory are described elsewhere (Rolls and Kesner, 2006; Rolls, 2008, 2010b; Kesner et al., 2012; Hunsaker and Kesner, 2013).

## Temporal order encoding and hippocampal function

There has for some time been evidence that the hippocampus plays a role in temporal order memory, even when there is no spatial component (Kesner et al., 2002; Rolls and Kesner, 2006; Hoge and Kesner, 2007). In humans, the hippocampus becomes activated when the temporal order of events is being processed (Lehn et al., 2009). An approach is now being developed on how temporal order memory could be implemented in the hippocampus (Rolls, 2010b, 2013; Rolls and Deco, 2010), as follows, and temporal pattern separation may be understood with this approach.

The approach is based on recent neurophysiological evidence of MacDonald, Eichenbaum and colleagues (MacDonald et al., 2011) showing that neurons in the rat hippocampus have firing rates that reflect which temporal part of the task is current. In particular, a sequence of different neurons is activated at successive times during a time delay period. The tasks used included an object-odor paired associate non-spatial task with a 10 s delay period between the visual stimulus and the odor. The new evidence also shows that a large proportion of hippocampal neurons fire in relation to individual events in a sequence being remembered (e.g., a visual object or odor), and some to combinations of the event and the time in the delay period (MacDonald et al., 2011).

These interesting neurophysiological findings indicate that rate encoding is being used to encode time, that is, the firing rates of different neurons are high at different times within a trial, delay period, etc. (Rolls and Deco, 2010; MacDonald et al., 2011). This provides the foundation for a new computational theory of temporal order memory within the hippocampus (and also the prefrontal cortex) which I outline next, and which utilizes the slow transitions from one attractor to another which are a feature that arises at least in some networks in the brain due to the noise-influenced transitions from one state to another.

First, because some neurons fire at different times in a trial of a temporal order memory task or delay task, the time in a trial at which an object (e.g., a visual stimulus or odor) was presented could become encoded in the hippocampus by an association implemented in the CA3 RCs between the neurons that represent the object [already known to be present in the hippocampus for tasks for which the hippocampus is required (Rolls et al., 2005; Rolls and Xiang, 2006)] and the ‘time encoding’ neurons in the hippocampus (MacDonald et al., 2011). This would allow associations for the time at which the object was present to be formed.

Second, these associations would provide the basis for the recall of the object from the time in a trial, or vice versa. The retrieval of object or temporal information from each other would occur in a way that is analogous to that shown for recalling the object from the place, or the place from the object (Rolls et al., 2002), but substituting the details of the properties of the “time encoding” neurons (MacDonald et al., 2011) for what was previously the spatial (place) component. In addition, if the time encoding neurons simply cycled through their normal sequence during recall, this would enable the sequence of objects or events associated with each subset of time encoding neurons to be recalled correctly in the order in which they were presented.

Third, we need a theory of what the origin is of the temporal effect whereby different hippocampal (or potentially prefrontal cortex) neurons fire in different parts of a trial or delay period. The properties of the “time encoding neurons” (Rolls and Deco, 2010; MacDonald et al., 2011) are key here, and we need to understand how they are generated. Are they generated within the hippocampus, or elsewhere, and in any case, what is the mechanism by which different neurons have high firing rates at different times in a trial? The fundamentally new approach to hippocampal function I am taking here is that rate encoding is being used, that is, the firing rates of different neurons are high at different times within a trial (Rolls and Deco, 2010; MacDonald et al., 2011). This is a radically different approach to order encoding than that based on phenomena such a theta and gamma oscillations that has been investigated by Lisman and colleagues (Lisman and Redish, 2009).

We can consider three hypotheses about how the firing of the ‘time encoding’ hippocampal neurons is produced. All utilize slow transitions between attractor states that can be a property of noisy attractor networks. The first hypothesis is that an attractor network with realistic dynamics (modeled at the integrate-and-fire level with a dynamical implementation of the neuronal membrane and synaptic current dynamics, and with synaptic or neuronal adaptation) can implement a sequence memory (Deco and Rolls, 2005). The hypothesis is that there are several different attractors, and that there are weak connections between the different attractors. In the model, adaptation produces effects whereby whatever sequence (order of stimuli) is presented on an individual trial, that order can be replayed in the same sequence because as one attractor state dies as a result of the adaptation, the next attractor to emerge from the spontaneous firing because of the spiking-related noise is the one that has been active least recently, as it is the one that is least adapted (Deco and Rolls, 2005). The whole system operates at a rather slow timescale for the transitions between the attractors partly because of the time for the noise to drive the system from one attractor state to another, and the slow time course of the adaptation (Deco and Rolls, 2005; Rolls and Deco, 2010). This implements a type of order memory.

The second hypothesis is analogous, and is also implemented in a recurrently connected system such as the hippocampal CA3 system or local recurrent circuits in the neocortex (Rolls and Deco, 2010). This second theory is that again there are several attractors, but that each attractor is connected by slightly stronger forward than reverse synaptic weights to the next. In previous work, we have shown that with an integrate-and-fire implementation with spiking noise this allows slow transitions from one attractor state to the next (Deco and Rolls, 2003; Deco et al., 2005). During learning of the synaptic weights in the network, adaptation might lead to each ‘time encoding’ population of neurons responding for only a limited period, helping to produce multiple sequentially activated populations of time encoding neurons (Rolls and Deco, 2010; MacDonald et al., 2011). In this scenario, an associative pool of neurons is unlikely to be helpful, and stronger forward that reverse weights between different attractors each consisting of a different population of ‘time encoding’ neurons would be the essence. It will be of interest to investigate whether this system, because of the noise, is limited to transitions between up to perhaps 7 ± 2 different sequential firing rate states with different neuronal subpopulations for each state, and thus provides an account for the limit of the magical number 7 ± 2 on short-term memory and related types of processing (Miller, 1956), and for the recency part of short-term memory in which the items are naturally recalled in the order in which they were presented. This is the most likely model at present of short-term memory and its natural propensity to store and to recall items in the order in which they were received (Rolls and Deco, 2010).

A variation on this implementation that I have proposed would be to have short-term attractor memories with different time constants (for example of adaptation), but all started at the same time (Rolls and Deco, 2010). This could result in some attractors starting early in the sequence and finishing early, and with other attractors starting up a little later, but lasting for much longer in time. The neurons recorded in the rat (MacDonald et al., 2011) are not inconsistent with this possibility. This type of time-encoding representation could also be used to associate with items, to implement an item-order memory.

It is thus suggested that temporal order memory could be implemented in the hippocampus in this way, and could make an important contribution to episodic memory in which several events linked in the correct order might form an episode. The theory shows how items in a particular temporal order could be separated from each other, a property we have referred to as the temporal pattern separation effect (Rolls and Kesner, 2006). The natural implementation of such temporal order memory would be in the hippocampal CA3-CA3 RC network. It is therefore somewhat of a puzzle that some of the evidence implicates the CA1 region in temporal order memory (Rolls and Kesner, 2006; Hunsaker et al., 2008; Kesner et al., 2012). This issue remains to be clarified. In any case, important in this temporal pattern separation would be the sparseness of the representation in the attractor, for if the representation became less sparse, this would impair the ability of the attractor network to maintain a long temporal sequence of different attractor states.

### Conflict of interest statement

The author declares that the research was conducted in the absence of any commercial or financial relationships that could be construed as a potential conflict of interest.
